# ConcreteXAI: A multivariate dataset for concrete strength prediction via deep-learning-based methods

**DOI:** 10.1016/j.dib.2024.110218

**Published:** 2024-02-20

**Authors:** José A. Guzmán-Torres, Francisco J. Domínguez-Mota, Elia M. Alonso-Guzmán, Gerardo Tinoco-Guerrero, Wilfrido Martínez-Molina

**Affiliations:** Civil Engineering Faculty, Universidad Michoacana de San Nicolás de Hidalgo, Morelia, Michoacán 58030, Mexico

**Keywords:** Artificial intelligence, Compressive strength prediction, Concrete properties, Mechanical tests, Non-destructive tests

## Abstract

Concrete is a prominent construction material globally, owing to its reputed attributes such as robustness, endurance, optimal functionality, and adaptability. Formulating concrete mixtures poses a formidable challenge, mainly when introducing novel materials and additives and evaluating diverse design resistances. Recent methodologies for projecting concrete performance in fundamental aspects, including compressive strength, flexural strength, tensile strength, and durability (encompassing homogeneity, porosity, and internal structure), exist. However, actual approaches need more diversity in the materials and properties considered in their analyses.

This dataset outlines the outcomes of an extensive 10-year laboratory investigation into concrete materials involving mechanical tests and non-destructive assessments within a comprehensive dataset denoted as ConcreteXAI. This dataset encompasses evaluations of mechanical performances and non-destructive tests. ConcreteXAI integrates a spectrum of analyzed mixtures comprising twelve distinct concrete formulations incorporating diverse additives and aggregate types. The dataset encompasses 18,480 data points, establishing itself as a cutting-edge resource for concrete analysis.

ConcreteXAI acknowledges the influence of artificial intelligence techniques in various science fields. Emphatically, deep learning emerges as a precise methodology for analyzing and constructing predictive models. ConcreteXAI is designed to seamlessly integrate with deep learning models, enabling direct application of these models to predict or estimate desired attributes. Consequently, this dataset offers a resourceful avenue for researchers to develop high-quality prediction models for both mechanical and non-destructive tests on concrete elements, employing advanced deep learning techniques.

Specifications Table

**Table:** 

Subject	Applied Machine Learning, Computational Materials Science, Civil and Structural Engineering.
Specific subject area	Analysis and prediction of concrete mechanical properties for civil infrastructure employing advanced regression methods, including machine learning algorithms.
Data format	Analyzed, Filtered
Type of data	Table, Text file
Data collection	Compressive, Flexural, and Tensile strength tests were conducted on the Forney Universal Machine. This electronic device has a 150-ton maximum load capacity of an approximation of 250 kg. The acquisition of data was established following the statements established on ASTM C 39-01, ASTM C-78-00, and ASTM C-496-96. Ultrasonic pulse velocity values were obtained using an electronic device of the brand CONTROLS, model E49, following the established criteria on the ASTM C597-02. Electrical resistivity parameters were captured using the Warner method and four-pin electrode type. The device is a resistor meter NILSSON, model 400. The procedure for acquiring the data was following the ASTM B193-20.
Data source location	• Institution: Materials laboratory “Ing. Luis Silva Ruelas” located at the Civil Engineering Faculty, Universidad Michoacana de San Nicolás de Hidalgo. • City: Morelia, Michoacán. • Country: México. • atitude and longitude (and GPS coordinates, if possible) for collected samples/data: 19.6896°N and 101.2039°E.
Data accessibility	Repository name: GitHub Data identification number: 10.5281/zenodo.10553470 Direct URL to data: https://github.com/JaGuzmanT/ConcreteXAI

## 1 Value of the Data

• The dataset includes concrete properties, mechanics, and non-destructive tests, which facilitates comprehensive exploration of material behavior. This is crucial for materials scientists and researchers aiming to understand the intricate relationships between various additives, materials, age testing, and cement brands. Machine learning algorithms can uncover patterns and correlations, leading to insights that may not be apparent through traditional methods.
• By leveraging the cutting-edge data in the dataset, machine learning models can be trained to predict concrete structures' structural performance and durability. Engineers and architects can benefit from these predictions to design safer and more resilient structures, optimizing construction practices based on empirical data.
• Machine learning algorithms applied to the dataset can contribute to robust quality control processes within the construction industry. Detecting patterns related to the influence of different additives, materials, and cement brands on concrete properties can guide manufacturers in optimizing their formulations for desired characteristics, ensuring consistent and high-quality construction materials.
• The dataset's richness in concrete properties and associated variables opens avenues for interdisciplinary collaboration. Researchers from various fields, such as materials science, civil engineering, data science, and machine learning, can leverage the dataset for their research needs. This interdisciplinary approach encourages ideas and methodologies, fostering innovation and enabling researchers to apply diverse perspectives to address complex challenges in concrete technology and construction.
• The diverse range of additives, materials, age testing, and cement brands in the dataset allows researchers to explore novel combinations and formulations. Machine learning can assist in identifying promising candidates for new concrete mixtures, leading to innovations in concrete technology. This is particularly relevant for creating sustainable and environmentally friendly construction materials. Also, the scope of applications of the proposed dataset is wide because it can be extended to different approaches that do not employ machine learning methods, for instance, concrete strength predictions.

## 2 Background

The motivation behind constructing this comprehensive dataset derives from the imperative need to advance our understanding of concrete properties and enhance the predictive capabilities in materials science and construction engineering. Recognizing the pivotal role of concrete in infrastructure development and the constant quest for improved materials, the dataset was designed to address existing gaps in knowledge.

The theoretical foundation for generating this dataset lies in materials science and structural engineering principles. It was essential to systematically capture the influence of various factors on concrete performance, including the types and proportions of additives, diverse materials, and variations across cement brands. This effort aligned to uncover hidden patterns and relationships that could contribute to optimized concrete formulations, improved structural design, and extended infrastructure lifespan.

Also, this dataset is valuable to extend and improve other methods that have been developed for concrete strength prediction without using machine learning techniques. Those methods are well explained and detailed in the state-of-the-art [1], [2], [3].

Methodologically, the dataset compilation involved meticulous documentation of mechanic and non-destructive test results across various conditions. Rigorous quality control measures were implemented to guarantee the reliability and accuracy of the dataset, aligning with the standards of materials science research and machine learning model training.

The ultimate aim was to provide a versatile tool for researchers and practitioners, fostering collaboration and contributing to advancements in concrete technology, construction practices, and infrastructure sustainability.

## 3 Data Description

The dataset described in this work comprises eleven columns. Each column has 4420 values, except the flexural and tensile strength columns because both of these contain fewer values.

The columns or attributes comprised in the dataset are the following:

• Type of cement
• Brand
• Additives
• Type of aggregates
• Design F'c (MPa)
• Curing age days
• Cs (Compressive strength) (MPa)
• Ts (Tensile strength) (MPa)
• Fs (Flexural strength) (MPa)
• Er (Electrical resistivity) (Ohm-cm)
• UPV (Ultrasonic pulse velocity) (m/s)

The dataset encompasses categorical and numerical values. The columns that handle categorical values are described in Table 1, and the numerical ones are enlisted in Table 2.

**Table 1:** Categorical columns and their corresponding information.

Column	Unique values	Number of values
Type of cement	‘CPO 30R RS BRA’, ‘CPC 40R’, ‘CPC 30R RS’, ‘CPC 30R’	4420
Brand	‘CEMEX’, ‘Apasco’	
Additives	‘Opuntia_ficus_indica’, ‘No_additions’, ‘Starch_fluidizer’, ‘fluidizer’, ‘Blast_furnace_slag_10%’	4420
Type of aggregates	‘Rounded’, ‘Crushed’, ‘Volcanic’ ‘Recycled’	4420

**Table 2:** Numerical columns and their corresponding information.

Column	Unique values	Number of values	Range of values
Design F'c (MPa)	25, 35, 30, 28	4420	[25–35]
Curing age days	3, 7, 14, 28, 40, 60, 120, 90	4420	[3–120]
Cs (MPa)	—	4420	[5.0823–55.7092]
Ts (MPa)	—	3460	[0.5311–5.2720]
Fs (MPa)	—	1760	[0.3367–8.0869]
Er (Ohm-cm)	—	4420	[1.8312–16.1693]
UPV (m/s)	—	4420	[2345.9812–4666.3548]

The dataset includes 4420 tests for the Cs, Er, and UPV, resulting in 13,260 values. Given the nature of building concrete prismatic specimens, the Fs column contains fewer values, storing 1760. On the other hand, Ts column contains 3460 values. So, all together, tests comprise a total of 18,480 continuous values.

### 3.1 Repository structure

**Fig. 1:**
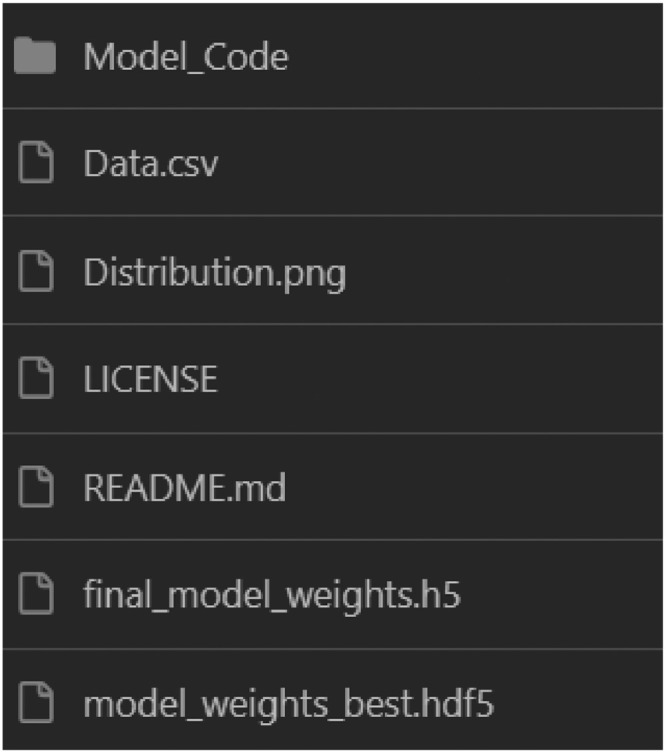
Root file of the repository.

**Fig. 2:**
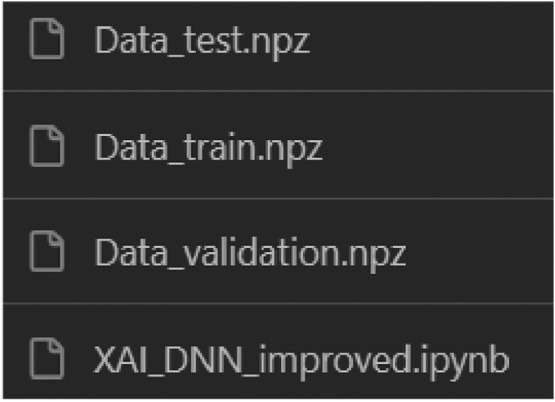
Files within the model_code folder.

The first view of the repository showcases a folder called “Model_Code”, which is represented in Fig. 1. In this folder, we found a Python script for reproducing a deep neural network to predict the concrete compressive strength parameter. Besides the deep neural network model, three datasets are within this folder, Data_train, Data_test, and Data_Validation in .npz extension (TensorFlow data type), all shown in Fig. 2.

**Fig. 3:**
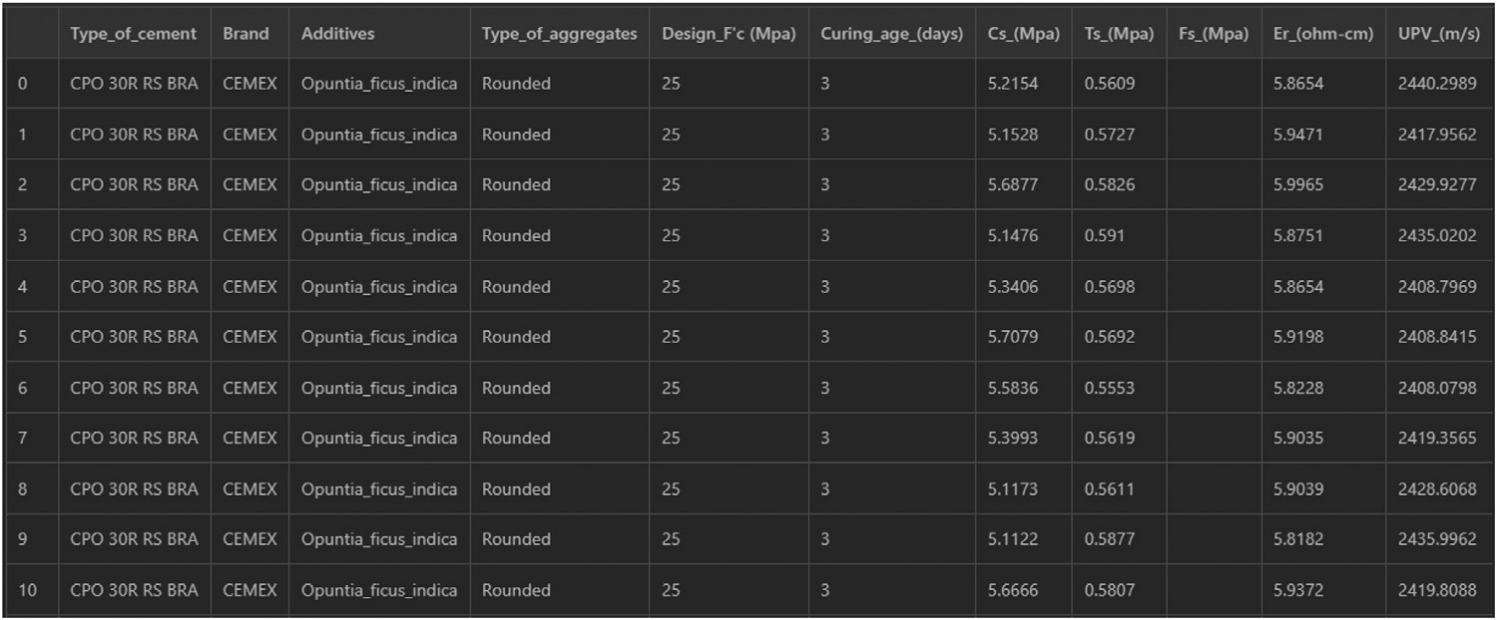
Dataset structure.

From the root file of the repository, we observed a text-plain file named “Data.csv”. This file contains the dataset of this manuscript and looks like the screenshot established in the Fig. 3.

The following files, Distribution.png, LICENSE, and README.md, encompass the repository's interface, providing a descriptive and suitable environment for the user. final_model_weights.h5 and final_model_weights.hdf5 are files that contain the weights of the trained deep neural network model localized in the folder Model_Code just ready to be used in other deep learning models or by leveraging the weights for using transfer learning techniques.

## 4 Experimental Design, Materials and Methods

First, and before the mechanical tests, we developed the non-destructive tests. These tests correspond to the Electrical resistivity and the ultrasonic pulse velocity. As a result of all the laboratory tests, we developed the Concrete XAI dataset.

In this work, two different kinds of concrete elements in shape, cylindrical and prismatic, were tested. The concrete cores (cylinders) measured 100×200 mm, and the prismatic ones had 150×150×600 mm dimensions.

For the electrical resistivity test, the Concrete XAI dataset adheres to relevant European standards, specifically EN 12390–Testing of hardened concrete. This standard ensures the accurate and standardized testing of concrete properties. Similarly, the ultrasonic pulse velocity test conducted in the dataset's development complies with European standards, particularly EN 12504–Testing concrete in structures. This standard provides a robust framework for evaluating concrete properties in structural applications. By incorporating compliance with these European standards, we aim to enhance the dataset's credibility, broaden its applicability, and ensure its relevance across diverse geographical contexts.

As a result of these combined efforts, the Concrete XAI dataset stands as a valuable resource, providing a comprehensive understanding of concrete materials through both mechanical and non-destructive assessments. The methodology developed for each test is described shortly.

### 4.1 Electrical resistivity data acquisition method

Electrical resistivity, a fundamental material property, is defined as the reciprocal of conductivity [4]. It is expressed in ohm-cm or ohm-m and is notably affected by pore saturation, paste hydration, and the presence of dissolved salts in the aqueous phase. Factors such as cement type, inorganic additions, water-cement ratio, and structural porosity further influence electrical resistivity [5].

The laboratory procedure for electrical resistivity measurement involves using concrete cores or extracted concrete elements. The necessary equipment includes:

1. Equipment for extracting cylindrical cores.
2. Electrical Resistivity Meter, utilizing the Wenner method with a 4-pin electrode type [6].
3. Equipment for measuring dimensions.

In cases where a resistivity measurement device is unavailable, the following can be utilized:

• Alternating current source or batteries.
• Voltmeter, with a reading capacity of 50 V, 1% precision, and high impedance (>10 MΩ).
• Milliampere meter, with a 0.1 to 250 mA capacity and 1% precision.
• Electrodes.

For our experiments, we employed the Nilsson brand Resistometer, a device specifically designed for measuring electrical resistivity. To minimize the influence of moisture, specimens were kept under minimal water flow until the test was conducted. The laboratory-level tests involve measuring dimensions (area, A, diameter, D, and length, L) and executing the test setup.

**Fig. 4:**
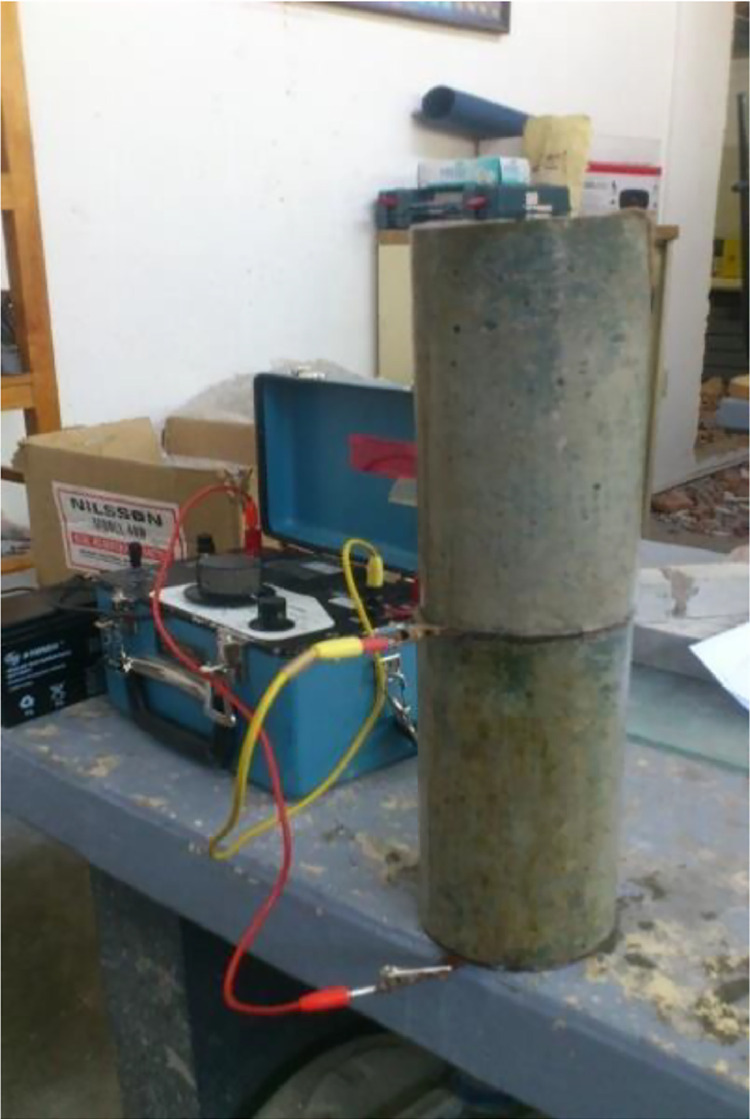
Electrical resistivity test in a concrete sample.

During the test, a given current (I) is applied to the specimen through metal plates attached to its lateral faces, and the resulting voltage (E) is recorded. The electrical resistance (*Re*) is calculated as E/I and expressed in ohms. The electrical resistivity (Er) is determined using Eq. (1), where A is the cross-sectional area of the specimen, L is the length of the specimen, *Re* is the electrical resistance, and Er is the electrical resistivity in ohm-cm (Ω-cm). Fig. 4 shows a visual representation of the electrical resistivity test conducted on a concrete cylinder using the resistance meter.

(1) $$Er\;=R_{e}\frac{A}{L},$$

where:

A = Cross-sectional area of the specimen.

L = Length of the specimen.

*Re* = Electrical resistance.

Er = Electrical resistivity in ohm-cm (Ω-cm).

### 4.2 Ultrasonic pulse velocity data acquisition method

The ultrasonic pulse velocity test is a crucial component of our study, providing insights into the mechanical properties of concrete. This non-destructive assessment is based on the relationship between the travel distance of an ultrasonic wave through concrete and the time it takes to traverse that distance [7].

The testing apparatus comprises a central unit that generates an electric pulse, transmitted to an emitter. The emitter excites a block of crystals, emitting an ultrasonic pulse through the concrete until detected by a receiver. The received ultrasonic pulse is then converted into an electric pulse, which is recorded on an oscilloscope. The time between the initial discharge and the pulse reception is electronically measured. By dividing the path length between transducers by the travel time, we obtain the average wave propagation velocity [8].

For this non-destructive test, the necessary equipment includes a couplant (such as silicone, Vaseline, or gel) and commercial ultrasonic equipment. Prior to testing, concrete specimens or test areas must have a flat, smooth surface free of dirt and should not be carbonated. Surfaces that are not sufficiently smooth should be regularized through mechanical processes or by applying a layer of cement paste, plaster, or epoxy resin with a minimum thickness to ensure good coupling with transducers without interfering with the measurement. Additionally, concrete specimens or test areas should exhibit homogeneity in composition and relative humidity.

To perform the test, the following steps are crucial:

1. Calibration. The device is calibrated using a reference bar or an equivalent device.
2. Transducer positioning. Depending on the testing mode, transducers are positioned as follows:
  • Direct Transmission: Transducers on opposite faces of the material.
  • Indirect Transmission: Transducers on the same face.
  • Semi-Direct Transmission: Transducers on adjacent faces.
3. Transducer placement: The transducer surfaces are placed and pressed onto the test area, and the setup is considered satisfactory when a minimum reading variation of ±1% is obtained.

In our study, readings of the ultrasonic pulse velocity test were specifically obtained through direct transmission. The wave propagation velocity or ultrasonic pulse velocity (UPV) is calculated using Eq. (2):

(2) $$UPV\;=\;\frac{d}{t},$$

where:

UPV = Wave propagation velocity (m/s).

d = Distance between coupling points (m).

t = Travel time from wave emission to reception (s).

**Fig. 5:**
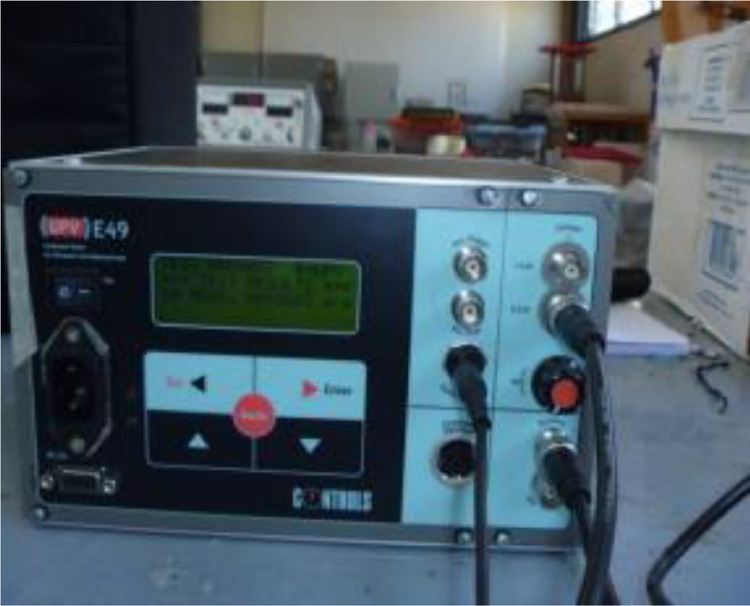
Electronic device for obtaining the ultrasonic pulse velocity on concrete specimens.

For visual reference, Fig. 5 displays the device used for applying the ultrasonic pulse velocity test.

### 4.3 Compressive strength data acquisition method

The aim of determining the uniaxial compressive strength of concrete is to evaluate its resistance and verify if it aligns with the project's design strength. This test is crucial for quality control, providing insight into the concrete's actual strength compared to the intended design strength [9].

To obtain the values of uniaxial compressive strength, the following equipment was utilized:

• Rule for measuring the cylinder diameter.
• Forney universal hydraulic testing machine.

The procedure for conducting this test is outlined below:

1. Determination of test specimen diameter. Measure the diameter of the test specimen with precision (±0.25 mm), averaging two perpendicular diameters.
2. Specimen placement. Place the specimen in the Forney universal hydraulic testing machine, ensuring the support plates are thoroughly cleaned. Center the vertical axis of the specimen on the support plate.
3. Adjustment of upper platen. Adjust the upper platen to the specimen face in a manner that minimizes impact load while making contact with the specimen.
4. Machine setup.
  • Level the machine and zero the readings.
  • Apply the load at a constant speed (continuous and impact-free) ranging from 0.137293 to 0.304006 MPa. The initial speed may be slightly higher during the first half of the total specimen load.
  • Avoid suspending load application and restart the process if needed before specimen failure.
5. Failure load determination. Predetermine the failure load by calculating the percentage of strength based on the specimen's age. Multiply the cross-sectional area of the cylinder by the design strength (F'c) [10].
6. Continuation until failure. Continue applying the load until failure occurs, recording the failure type and the appearance of the material.

It is crucial to conduct compression tests on specimens cured in a wet environment promptly after removal from the curing facility, once the curing material has achieved the required strength.

To calculate the actual stress that the concrete can withstand, divide the resistant force (P) by the cross-sectional area (A) using Eq. (3):

(3) $$Cs=\frac{P}{A},$$

where:

P = Failure load in kg.

A = Cross-sectional area of the specimen in cm².

Cs = Compressive strength that the specimen can withstand in MPa.

**Fig. 6:**
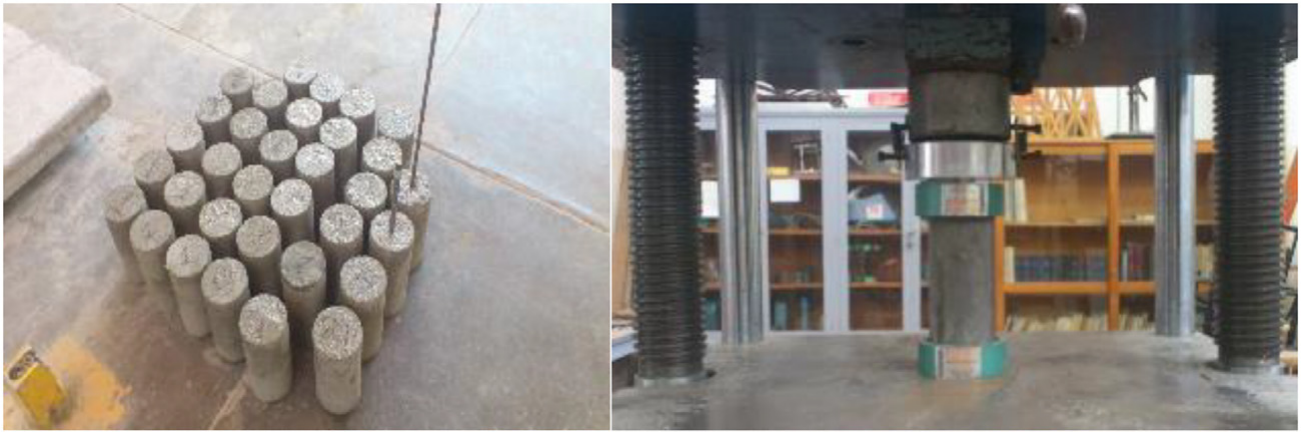
Concrete elements and the compressive strength test.

Some concrete specimens and the compressive strength test developed on the Forney universal hydraulic testing machine are shown in Fig. 6.

### 4.4 Tensile strength data acquisition method

This section outlines the methodology employed for acquiring data to determine the indirect tensile strength of concrete cylinders. The tensile strength is a crucial parameter for understanding concrete behavior and presents challenges in concrete design due to the influence of various parameters on failure criteria [11]. To ensure comprehensive understanding, this section now provides a detailed description of the experimental design and methods, including tools, instruments, and experimental conditions.

### 4.5 Equipment used

The mechanical tests were conducted using the following equipment:

• Forney universal hydraulic testing machine.
• Supplementary support plate or bar.
• Plywood support strips.

Each specimen utilized two plywood support strips, measuring 3 mm (1/8″) in thickness, free of imperfections, with a width of 25 mm (1″) and a length equal to that of the specimen. These strips are single-use, and specimens were maintained in a humid environment throughout the testing process.

### 4.6 Test procedure

The procedure for obtaining data from the indirect tensile strength test is detailed as follows:

1. Specimen marking. Diametrical lines were marked at each end of the specimens using a suitable device to ensure they lie within the same axial plane.
2. Dimension measurements. Diameter dimensions were obtained by averaging measurements near the ends and one at the center along the diametrically marked plane. Length was measured by averaging two measurements of lines connecting the ends of each diameter-marked line on both faces of the specimen.
3. Plywood Strip Placement. One plywood strip was placed along the center of the lower support plate. The specimen was positioned on the plywood strip, vertically aligning the marked lines on its two ends and concentrating them on the strip. The second plywood strip was placed longitudinally over the cylinder, focusing on the marked lines at the ends. The assembly ensured that the extension of the plane containing the two marked lines at the ends of the specimen passes through the center of the upper support plate. When used, the specimen's supplementary plate and center were directly beneath the center of the spherical support plate.
4. Application of Load. The load was applied continuously and without impact at a uniform rate within the range of 0.6864 to 1.3729 MPa of indirect tensile stress until specimen failure. The maximum applied load at failure, the type of failure, and the appearance of the concrete were recorded [12].
5. Calculation of indirect tensile strength. The indirect tensile strength (Ts) was calculated using Eq. (4).
  (4) $$Ts=\frac{2P}{\left(dL\pi \right)},$$

where:

Ts = Indirect tensile strength in MPa.

P = Maximum applied load in kg.

L = Length in cm.

d = Diameter in cm.

**Fig. 7:**
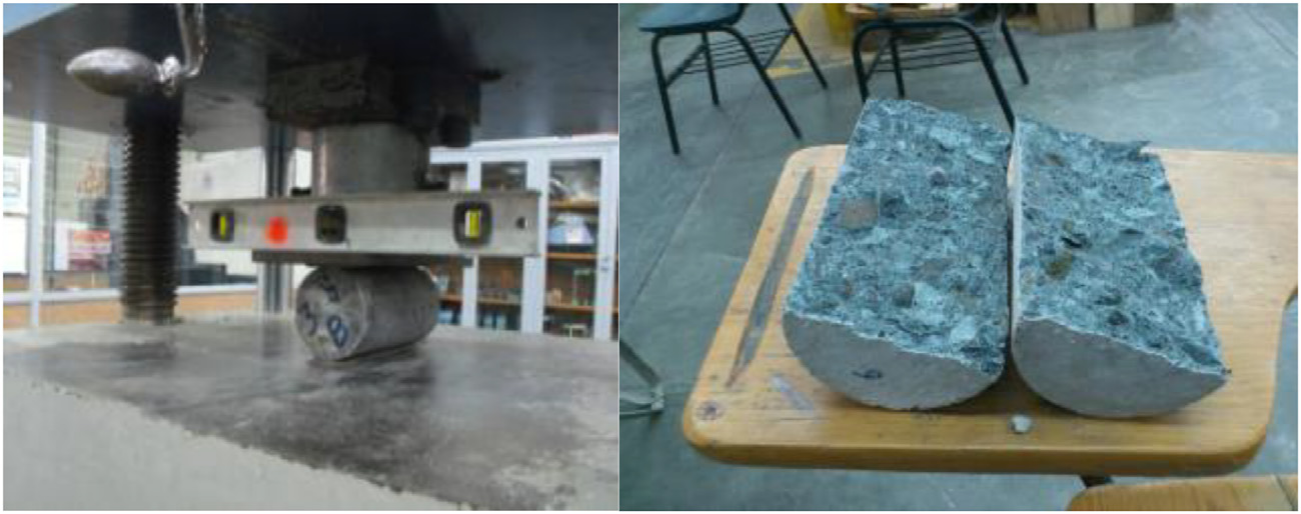
A concrete element tested to indirect tensile strength and its failure after applying the load.

In Fig. 7, it is possible to see the indirect tensile strength in a concrete element and its respective failure.

### 4.7 Flexural strength data acquisition method

To determine the flexural strength of concrete, the first step involved the creation of concrete beam specimens. This process required the utilization of either a Forney universal hydraulic testing machine or a hydraulic testing machine, along with specific flexural accessories.

Concrete specimen preparation followed a meticulous set of steps:

1. Positioning.
  • The specimen was turned onto one of its sides from its original casting position.
  • Marking of positions for the four supports was done using a crayon.
2. Alignment.
  • We ensured proper alignment of the lower supports, ensuring that the upper supports made contact with the specimen's upper face at the external points of the central third of the span between the inner supports.
3 Surface quality check.
  • In cases where good contact with the inner supports was not achieved, the specimen might undergo polishing, headering, or shimming with wood or steel strips to improve contact surfaces.
4. Load application.
  • Application of the load was done uniformly to avoid impact, starting rapidly until just under 50% of the rupture load.
  • The load was then applied so that the stress on the outer fiber did not exceed 10 kg/cm²/min (980 kPa/min).

5. Measurement.
  • Dimensions of the specimen, including the average width and depth at the failure section, were measured, rounding measurements to the nearest 0.25 cm.

Calculations were conducted based on the location of the fracture:

If the fracture occurred in the middle third of the span [13], the modulus of rupture (Fs) was calculated using Eq. (5):

(5) $$Fs=\frac{3PL}{2bd^{2}}.$$

If the fracture occurred outside the middle third but within 5% of the span, the modulus of rupture was calculated with Eq. (6):

(6) $$Fs=\frac{PL}{2bd^{2}},$$

where:

Fs = Modulus of rupture in MPa.

P = Rupture load in kg.

L = Span in cm.

b = Average width in cm.

*d* = Average depth in cm.

**Fig. 8:**
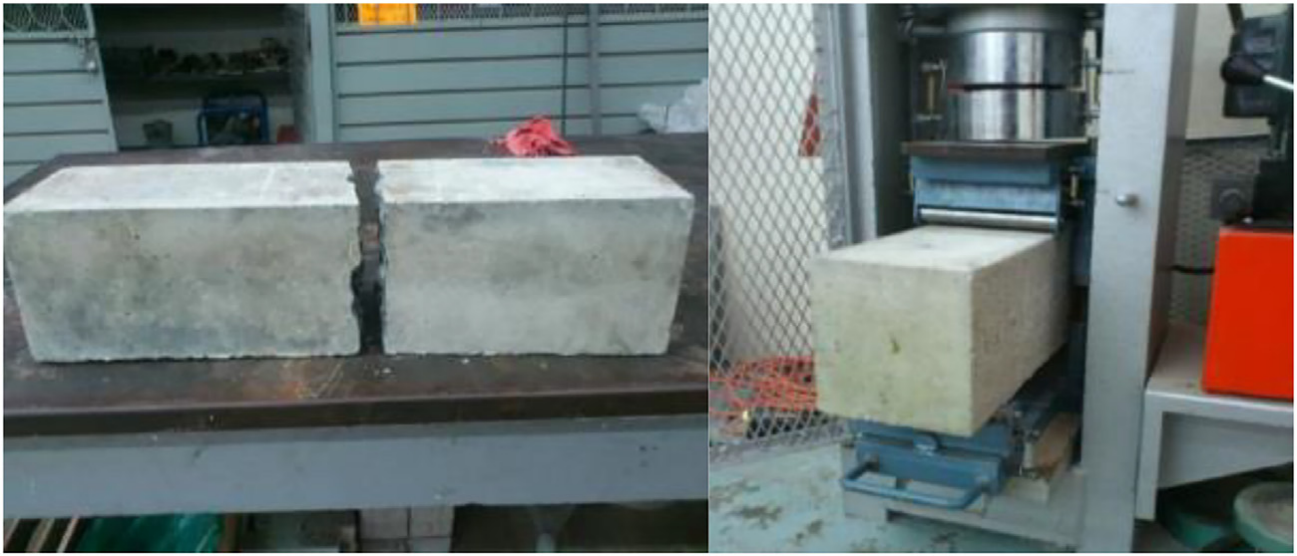
Failure on concrete beam tested to flexural strength and its implementation.

It is crucial to note that results must be discarded if the fracture occurs outside the middle third in more than 5% of the span. All flexural strength results were calculated using Eq. (5), considering the type of failure in the concrete beams. Refer to Fig. 8 for a visual representation of a tested concrete beam and the hydraulic testing machine used for obtaining the Fs data.

## Limitations

The acquisition of data in all the tests was satisfactory. However, there is a couple of tests (*T*s and Fs) where the amount of data is lower due to the complexity of the test and the quantity of materials that the concrete specimens shape requires to be elaborated. All the tests were performed under controlled laboratory conditions, though there could be slight variations in the distribution of data because of human errors.

## Ethics Statement

The authors of this dataset meet the ethical requirements for publication in Data in Brief and do not involve human subjects, animal experiments, or any data collected from social media platforms.

## CRediT authorship contribution statement

**José A. Guzmán-Torres:** Conceptualization, Methodology, Writing – review & editing, Data curation, Visualization, Investigation. **Francisco J. Domínguez-Mota:** Visualization, Supervision. **Elia M. Alonso-Guzmán:** Investigation, Writing – review & editing. **Gerardo Tinoco-Guerrero:** Methodology. **Wilfrido Martínez-Molina:** Investigation, Writing – review & editing.

## Data Availability

ConcreteXAI (Original data) (GitHub).
